# Multi-agent simulation model updating and forecasting for the evaluation of COVID-19 transmission

**DOI:** 10.1038/s41598-022-22945-z

**Published:** 2022-12-21

**Authors:** Brenno Moura Castro, Marcelo de Miranda Reis, Ronaldo Moreira Salles

**Affiliations:** Military Institute of Engineering (IME), Pc General Tibúrcio 80, 22290-270 Rio de Janeiro, Brazil

**Keywords:** Immunology, Environmental social sciences, Diseases, Health care, Health occupations, Risk factors, Mathematics and computing

## Abstract

Agent-based models have been an emerging approach in epidemiological modelling, specifically in investigating the COVID-19 virus. However, there are challenges to its validation due to the absence of real data on specific socio-economic and cognitive aspects. Therefore, this work aims to present a strategy for updating, verifying and validating these models based on applying the particle swarm optimization algorithm to better model a real case. For such application, this work also presents a new framework based on multi-agents, whose significant contribution consists of forecasting needed hospital resources, population adaptative immunization and reports concerning demographic density, including physical and socio-economic aspects of a real society in the modelling task. Evaluation metrics such as the data’s Shape Factor (SF), Mean Square Error (RMSE), and statistical and sensitivity analyses of the responses obtained were applied for comparison with the real data. The Brazilian municipality of Passa Vinte, located in the State of Minas Gerais (MG), was used as a case study. The model was updated in cumulative cases until the 365th day of the pandemic. The statistical and sensitivity analysis results showed similar patterns around the actual data up to the 500th day of the pandemic. Their mean values of SF and RMSE were 0.96 and 7.22, respectively, showing good predictability and consistency, serving as an adequate tool for decision-making in health policies.

## Introduction

Multi-agent-based models have aroused the interest of many researchers in several applications. Its approach consists of a method for analyzing “complex social systems” characterized by multiple interacting parts and may present non-linear behaviours.

Policymakers and researchers have applied these models exhaustively, seeking answers to epidemiologic and biological issues. For instance, to investigate the effects of a given pathogen on society and the human body, experimental testing conditions would put the safety of people at risk, creating ethical problems and/or biological disasters. Thus, multi-agent models consist of autonomous agents who work toward goals (as opposed to discrete tasks) in a dynamic environment (where change is the norm), without continuous direct supervision or control, and exhibit a significant degree of flexibility^6^.

Therefore, the multi-agent approach becomes a reasonable and suitable proposal for modelling due to the variety of effects caused by the Severe Acute Respiratory Syndrome-2 (SARS-CoV-2) virus in the human body and society Corona Virus Disease (COVID-19) spreading. However, it is worth mentioning the following question: how may these models be evaluated and validated?

Using multi-agent models to model real-world and social systems has attracted criticism due to the challenges involved in their validation^20^. This work studied an intra-state conflict multi-agent-based model to give a balanced perspective on those criticisms. Indeed, multi-agent models for social systems are most useful when the connection between micro-behaviours and macro-behaviours is not well-understood. Another point is when data collection from the real-world system is prohibitively expensive for time or money or if it puts human lives at risk.

Despite the criticisms regarding agent-based models, this approach is advantageous when agents are heterogeneous, and the heterogeneity of the agents affects the overall performance of the system. Also, these models provide more detailed information than equation-based models or many other approaches. These agents’ interactions are frequently non-linear, changing both spatially and temporally. The emphasis is on modelling, capturing, and replicating new emergent properties in a complex system of individual components that evolve and interact^2,11,24,30^. Therefore, it is often easier to think about the individual level than the aggregate level for many phenomena, especially social phenomena^30^.

The multi-agent models for social systems are challenging to validate due to the absence or difficulty of finding population-specific and cognitive real data, which are crucial to achieving a reliable degree of veracity. For instance, supposing an epidemiological model, relationships and behaviours of individuals are believed to influence the spread of diseases. Such aspects should be modelled from extended and costly data collecting. However, the lack of data does not mean that we cannot try to understand the dynamics of a system^20^.

As an example of a real application, the work^27^ approached an agent-based model to investigate the dynamics and transmission of COVID-19 among the inhabitants of a city, specifically in Ford County, KS, USA and New York City, USA. In the last case, a scaled-down model version was used. In addition, the approached model could be adapted for any realistic scenario by incorporating appropriate parameters specific to the city under consideration.

Another example can be seen in the work^23^ regarding the study of the effective evacuation of buildings, especially a school drill. Both works^23,27^ are validated by comparison of the real data and model results using error and evaluation metrics between real data and model results.

Other validation processes based on the application of gradient-based algorithms and performance evaluation of the results obtained through out-of-sample prediction errors can be seen in the works^1,10,25^ regarding the financial stock market analyses.

According to work^9^, analyzing historical data is the more prevalent method for updating agent-based models. The historical data method is more practical due to its excellent fit and easy verification. The historical data is split into collected data for modelling or update tasks and a verification set to evaluate the model and verify results. In other words, it consists of carried out by visually and statistically evaluating the model’s ability to replicate (qualitatively and quantitatively) some real data or stylized facts^3,4,17^.

Many previous works addressed the literature’s procedures and tasks for verification and validation processes. For example^29^, proposed a significant steps roadmap for the validation process based on real data whose purpose is to be used prescriptively. Moreover, some works aimed to demonstrate specific techniques within roadmap steps, such as input parameters’ sensitivity analysis, statistical analysis and others^12,16,18,22,26^.

Recently, other validation techniques have become emergent, such as using artificial intelligence (AI) and metaheuristic algorithms. The work^28^ introduced Reinforcement Learning for calibrating the parameters based on the existing state transfer equations that link the micro-parameters and macro-states of the multi-agent system. As a case study, it was used in a population migration model. Similarly, these approaches can be seen in the works^8,13,14,31^, for example.

Therefore, according^2^, agent-based models have proven to be effective as a tool for integrating knowledge across stakeholders in order to solve management problems, understand co-evolution and emergent phenomena, and address adaptive management issues to date.

As multi-agent models have been an emergent approach in pandemic modelling, a growing concern has arisen about how to validate such models. Therefore, this work aims to present an update, verification and validation strategy based on applying the particle swarm optimization algorithm to define the multi-agent model’s sensitive parameters that best represent the modelling of a real spreading case of the SARS-CoV-2. Evaluation metrics such as shape factor (SF) of the data curves, mean squared error (RMSE) and statistical and sensitivity analyses of the responses obtained were applied for comparison and evaluation with the real data. The Brazilian municipality of Passa Vinte, located in the State of Minas Gerais (MG), was used as a real case study.

For this purpose, the epidemiological multi-agent-based *MCovidSim-2* framework is proposed in this work consists of some improvements to the model discussed in the work^6^. The significant contribution of this new framework consists of forecasting needed hospital resources, population adaptative immunization and reports concerning demographic density in regions such as hospitals, industrial and commerce, schools and circulation areas, including physical and socio-economic aspects of a real society in the modelling task. Moreover, this model allows modelling the immunity gained due to agents’ recoveries and reinfections over model interactions. Therefore, these improvements can suit as tools for public health managers concerning society policy applications and medical resources’ management (professionals, equipment, medicines and others).

This paper outlines the epidemiological multi-agent-based *MCovidSim-2* framework implementation, which discusses these improvements in “Epidemiological multi-agent framework: *MCovidSim-2*”. The description of an updating, verification and validation strategy using the particle swarm optimization algorithm and evaluation metrics is given in “Updating, verification and validation strategy”. “Case study” describes the case study using real data from the Brazilian municipality Passa Vinte, located in the Minas Gerais (MG) State. Updating, verification and validation tasks were described in “Results and discussions”. Also, this section presents a sensibility and statistical analysis of the obtained results from various solutions of the updated model concerning the forecasting of pandemic evolution, hospital resources and agents’ weekly density in each region of the model environment. Concluding remarks are given in “Conclusion and recommendations”.

## Epidemiological multi-agent framework: *MCovidSim-2*

The work^6^ proposed a framework $M^2CovidSim$ multi-agent-based framework to investigate the spread of COVID-19 simulating hypothetical social scenarios as a case study. Thus, this paper brings some improvements in the *MCovidSim* to implement real and peculiar aspects observed during the evolution of the pandemic. The new version of this framework proposes new states of the agents, environment setup and demographic density in *MCovidSim-2*.

**Figure 1:**
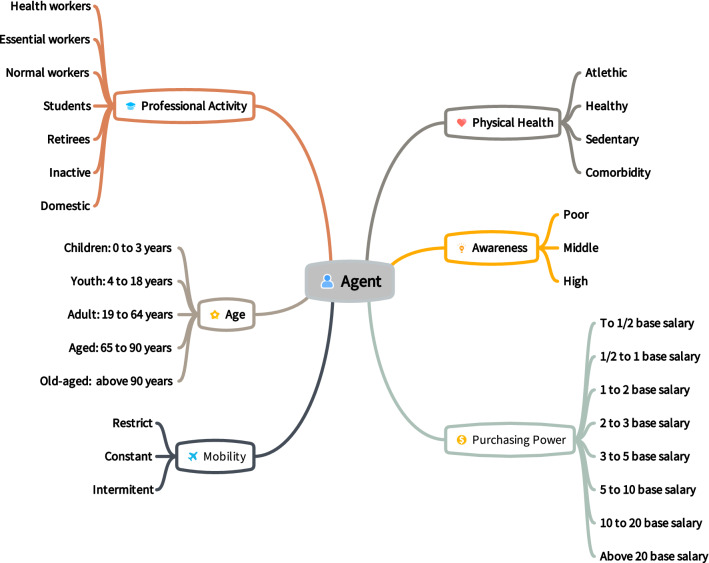
Set up the physiological and socio-economic aspects of the agent to detail the matrix that stores the agent’s attributes in the model.

Similarly, *MCovidSim-2* describes that each agent has its physiological and socio-economic characteristics based on works^6,19^ described in Fig. 1. Thus, it was assigned a scale from 0 to 100^19^ for the scores according to the characteristics attributed to the agents to calculate the following aspects of each agent: (i) probability of contracting the disease ($\beta$); (ii) recovery time ($T_{rec}$); (iii) probability of death ($\gamma$) and (iv) rules of movement. Further details can be seen in the work^6^.

Similarly to the approach in^6^, this work uses the range of values established by the works^7^ to calculate $\beta$, $T_{rec}$, $\gamma$ and rules of movement. The probability of hospitalization ($\delta$) and the time of immunization ($T_{im}$) of each agent is calculated using the following equations for *n*th agent:

1 $$\begin{aligned} \delta _n= & {} \frac{\text {physical health(n)} + \text {purchasing power(n)}}{2} \end{aligned}$$

2 $$\begin{aligned} T_{im_n}= & {} \frac{\text {physical health(n)} + \text {awareness(n)}}{2} \end{aligned}$$

### Implemented rules and environment’s setup

Therefore, each agent may have one of the seven states: (i) susceptible; (ii) infectious; (iii) infectious under hospitalization; (iv) infectious without hospitalization (due to insufficiently hospitalization available resources); (v) recovered with immunization; (vi) recovered without immunization or; (vii) deceased.

**Figure 2:**
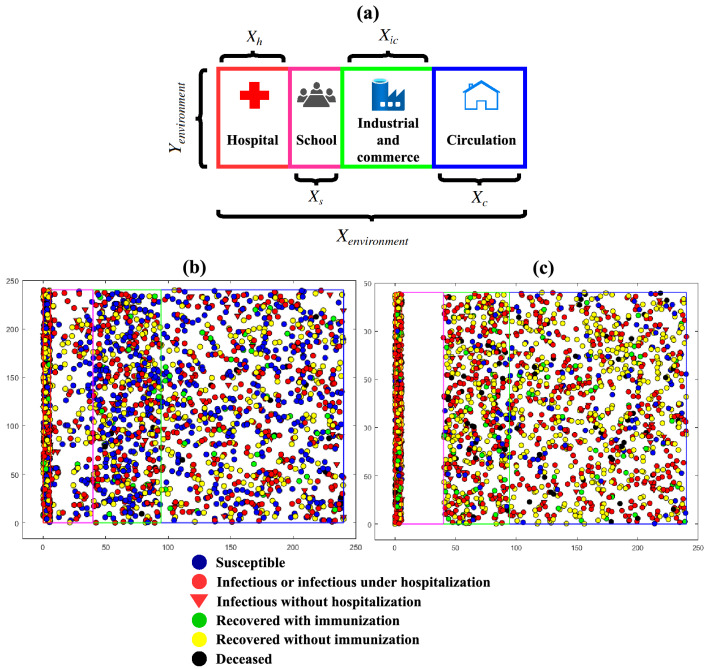
Example of environment model: (**a**) setup of areas; (**b**) without social distancing and (**c**) with social distancing.

Unlike the previous version, the proposed framework seeks to contribute to analysing the demographic density over simulation time iterations. For this reason, the environment is set up to represent the demographic density per specific region. In other words, the model environment’s extensions do not represent the real dimensions of a studied region. This implementation aims to circumvent the difficulty of measuring real extensions of a city’s specific areas and regions. Thus, medical facilities and hospitals, schools, industrial and commerce, and circulation areas are defined as a percentage of the entire environment’s horizontal dimension (x-axis), as shown in Fig. 2a.

In addition, Fig. 2b,c present an example of an environment model without social distancing and with social distancing and kinds of model agents.

$M^2CovidSim$-2 is composed of three main sets of rules: (1) rules regarding possible agents’ states (*susceptible*, *infected*, *infected with hospitalization*, *infected without hospitalization*, *recovery with immunization*, *recovery without immunization* and *deceased)*; (2) Rules implementing the contagious probability evolution of the agents; (3) Rules of the agent’s movement in the simulation environment according to its respective state.

**Figure 3:**
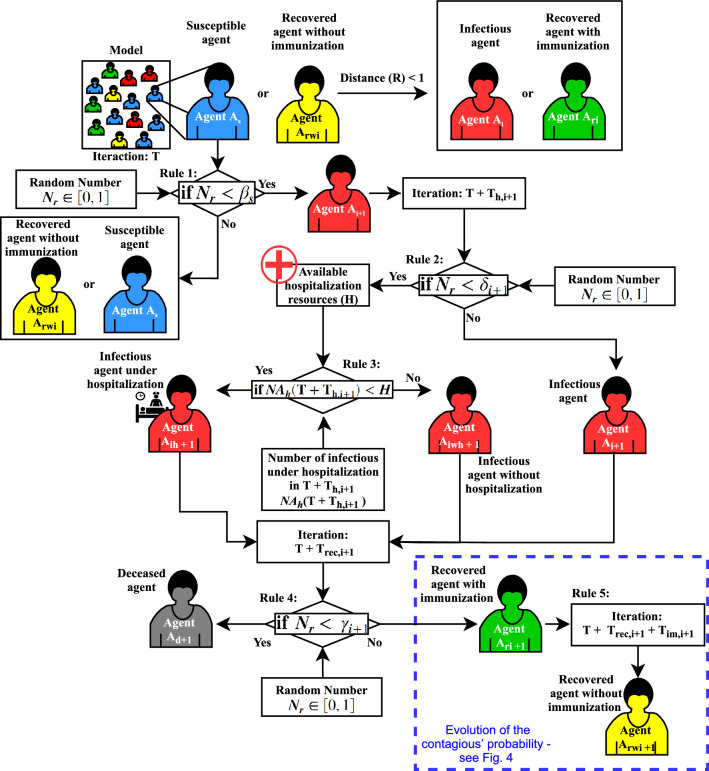
Roadmap of applying the agents’ infect, recover and decease evolution rules implemented in the *MCovidSim-2* framework.

**Figure 4:**
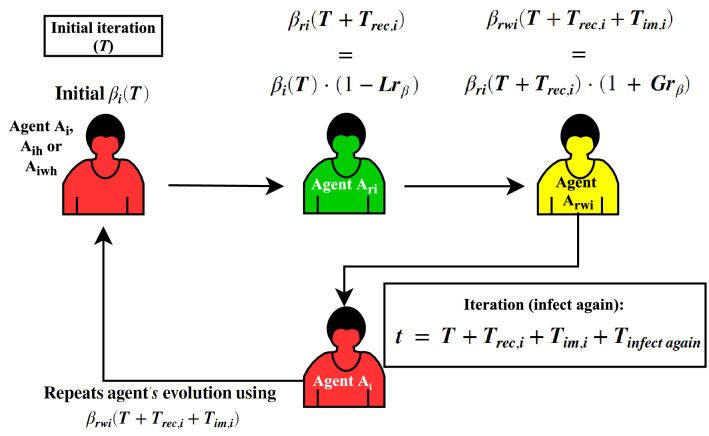
Roadmap of evolutions of the contagious’ probability ($\beta$) over time model interactions.

Therefore, Fig. 3 describes the roadmap implementation concerning the set of rules to possible agents’ states. Figure 4 presents the roadmap of evolutions of the contagious’ probability ($\beta$) over time model interactions.

Note that rule no. 3 described in Fig. 3 represents the contribution of this framework regarding evaluating and forecasting the chosen city hospital’s capacity to assist its population in the face of the pandemic. Additionally, this framework also contributes to analysing the immunological adaptation of each agent over the simulation time iterations. This consideration is described in rule no. 5 (Fig. 3) and the roadmap shown in Fig. 4.

As described, the procedures of the virus spreading, possible agents’ states and evolution of the contagious’ probability (Figs. 3, 4), as follows, it is described the agents’ rules of movement inside the environment’s areas presented in Fig. 2.

### Agents’ movement rules

**Figure 5:**
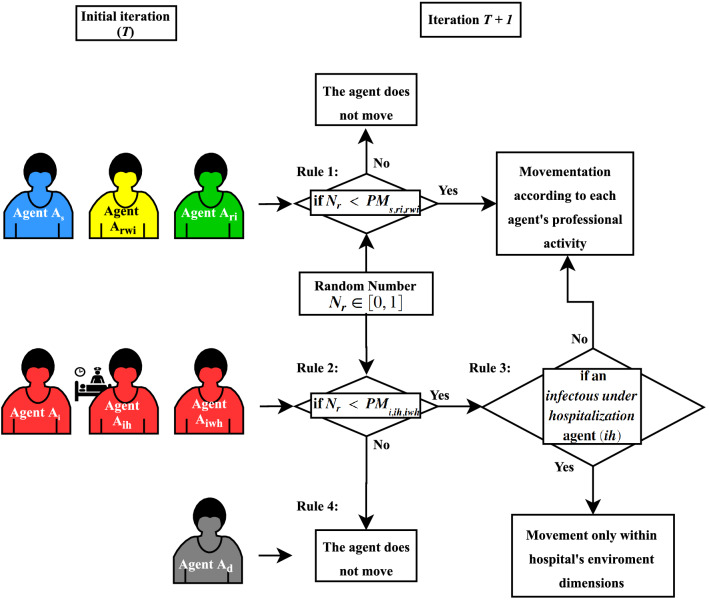
Roadmap of the Movement’s rules.

The multi-agent-based framework is based on four agent movement rules. Each rule is intrinsically related to the agent’s state. Equation (3) describes the movement probability concerning the agent’s state classified as: *susceptible*, *Recovered with immunization* and *Recovered without immunization*. Meanwhile, Eq. (4) describes the movement probability concerning the *Infectous*, *Infectous under hospitalization* and *Infectous without hospitalization*. Otherwise, deceased agents remain static within the entire environment over model time iterations. Figure 5 shows the movement’s rules roadmap.

3 $$\begin{aligned} PM_{s,ri,rwi}= & {} \frac{\text {mobility}_{s,ri,rwi}}{100} \end{aligned}$$

4 $$\begin{aligned} PM_{i,ih,iwh}= & {} \frac{\text {mobility}_{s,ri,rwi}\cdot \text {awareness}_{s,ri,rwi}}{100} \end{aligned}$$

According to^6^, a movement indicates that an agent will switch regions. If an agent moves, the algorithm generates a random number from 1 to 4. This number represents the four existing regions in the environment: (1) circulation area, (2) commercial and industry, (3) hospitals, and; (4) schools. The function that generates this random number uses the restrictions imposed for an agent to be in one of the specified regions, determined by its professional activity and the social distancing policy.

## Updating, verification and validation strategy

One of the criticisms of using multi-agent models to study social systems is to there is often a paucity of data from relevant real-world social systems. Consequently, it becomes difficult to both ground the model in reality and validates its output^20^. In other words, the measured and quantify data concerning the agents’ cognitive aspects such as awareness, physical health, and mobility are not found in literature and government institutes.

In short, collecting data about a social system may be complex for several reasons. Nevertheless, the lack of these data does not mean that we cannot try to understand how a system may behave systematically and formally. Thus, multi-agent models could represent what are believed to be the essential structures and variables and then fill in the data that are too costly to collect from the real world.

Then, it can suppose that these agents’ parameters are the most sensitive within the proposed framework modelling. Also, over time model iterations, Fig. 4 describes the evolution of each agent’s probability of contracting the disease ($\beta$) evolution. Thus, rate of loss ($Lr_{\beta }$) and rate of growth ($Gr_{\beta }$) concerning $\beta$ also can be sensitive parameters.

Additionally, concerning the environment, it is also possible to suppose that the definition of the entire X-axis dimension and areas’ extensions, described in Fig. 2a, are sensitive parameters due to demography density modelling. In other words, it can assume that *MCovidSim-2* has eleven sensitive parameters.

Therefore, the approached strategy for updating, verification and validation consists of an optimization algorithm’s application to seek a parameters setup described before that better represents the real data of cumulative cases and cumulative deceased in a given modelled region, city and municipality.

For this work, it was used particle swarm algorithm^15^ to seek better parameters. The objective function is defined by the ratio between RMSE (calculated from comparing real data and model results—Eq. (5)) and the SF (Eq. 6). The SF evaluates the degree of consistency/similarity between real data and model results curves. It varies from 0 to 1 (0 stands for no consistency/similarity, and; 1 stands for consistency/similarity of the curves). In these equations, $x_m(t)$ and $x_r(t)$ represent model and real data, respectively, about cumulative cases curves and; *s* denotes the number of the curves’ samples.

Table 1 describes the sensitive parameters threshold and constraints of the optimization problem.

5 $$\begin{aligned} RMSE(x_m,x_r)= & {} \sqrt{\sum ^{s}_{j=1}\frac{(x^{j}_{r} - x^{j}_{m})}{s}} \end{aligned}$$

6 $$\begin{aligned} SF(x_m,x_r)= & {} \frac{|x_m \cdot x_r|^2}{(x^{T}_{r}\;x_r) \cdot (x^{T}_{m}\; x_m)} \end{aligned}$$

**Table 1:** Sensitive parameters threshold and constraints of the optimization problem.

Sensitive parameters’ threshold (supposing model iterations in days)			
$\beta$	$\in$	$[0.01\;\;0.50]$	−
$T_{rec}$	$\in$	$[10\;\;200]$	days
$T_{im}$	$\in$	$[15\;\;200]$	days
$\gamma$	$\in$	$[10^{-4}\;\;10^{-2}]$	−
$Lr_\beta$	$\in$	$[0.01\;\;0.80]$	−
$Gr_\beta$	$\in$	$[0.01\;\;0.70]$	−
$X_{environment}$	$\in$	$[50\;\;1000]$	–
$X_h$	$\in$	$[1\;\;10]$	%$X_{environment}$
$X_s$	$\in$	$[1\;\;20]$	%$X_{environment}$
$X_{ic}$	$\in$	$[1\;\;40]$	%$X_{environment}$
$X_c$	$\in$	$X_{environment} - X_h - X_s - X_ic$	%$X_{environment}$
**Constraints**			
$Lr_\beta \,>\,Gr_\beta$	Human body’s adaption to the pandeimc		
$X_h + X_s + X_{ic} < 0.30 \cdot X_{environment}$	Minimal circulation area dimension		

The objective function is defined according to as follows:

7 $$\begin{aligned} min \;\frac{{\mathrm {RMSE}\left( x_m \;,x_r \right) }_{\mathrm {infected}} \cdot {RMSE\left( x_m ,x_r \right) }_{\mathrm {deceased}} }{{SF\left( x_m ,x_r \right) }_{\mathrm {infected}} \cdot {SF\left( x_{m,} x_r \right) }_{\mathrm {deceased}} } \end{aligned}$$

Case there are no deceased individuals in the real data:

8 $$\begin{aligned} min \;\frac{{\mathrm {RMSE}\left( x_m \;,x_r \right) }_{\mathrm {infected}} }{{SF\left( x_m ,x_r \right) }_{\mathrm {infected}} } \end{aligned}$$

**Figure 6:**
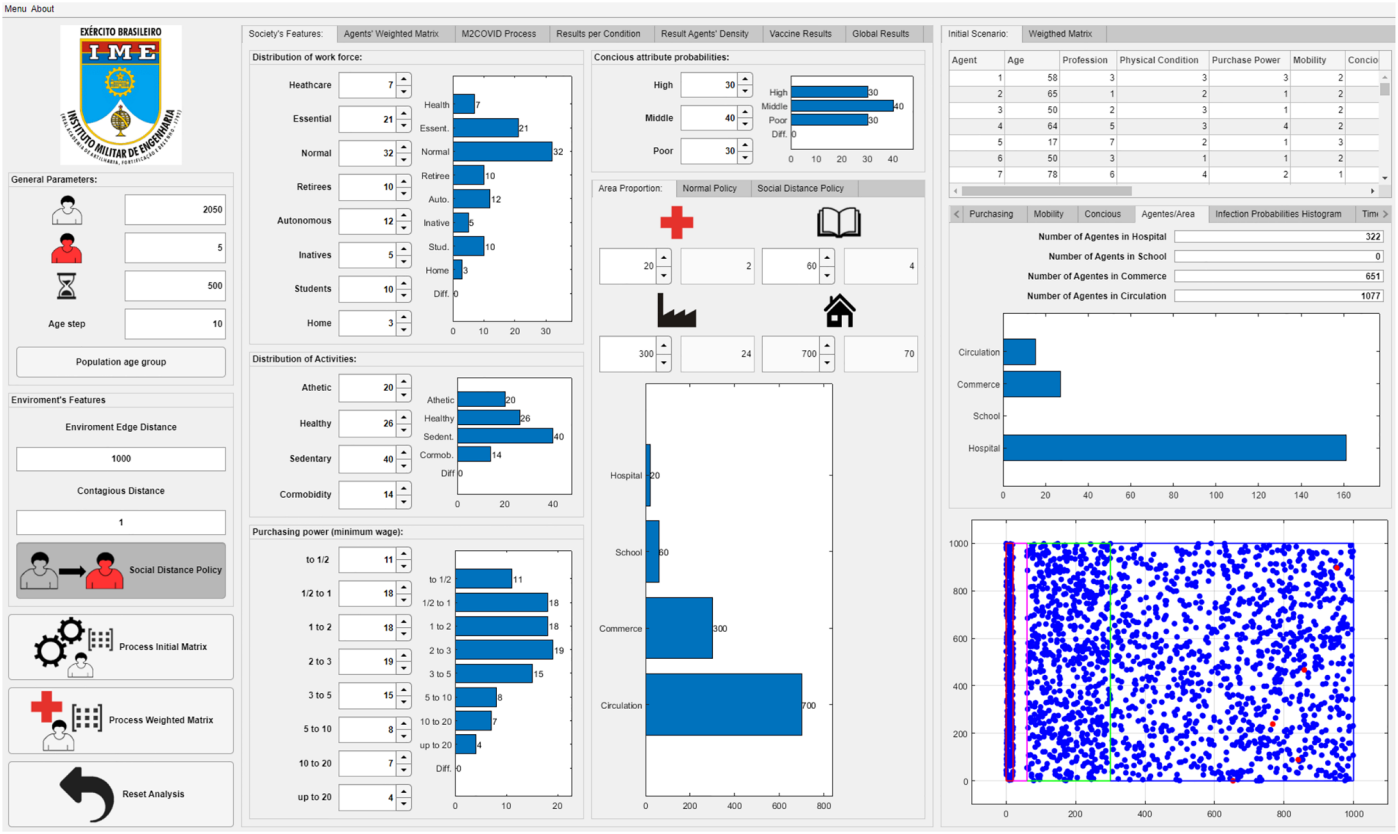
*MCovidSim-2* framework.

Thus, defining values of the sensitive parameters, *MCovidSim-2* was processed thirty times, obtaining model results concerning accumulated infected and deceased curves. This task aims to provide a statistical analysis of the responses from the updated *MCovidSim-2*. Figure 6 presents the *MCovidSim-2* framework.

## Case study

The work^16^ mentions that the multi-level property of agent-based simulation is the size of the model. Therefore, the input parameters-output response relations may be multi-dimensional and rich. Consequently, the multi-agent-based models’ updating, verification, and validation tasks are not trivial and require relevant computational costs. Therefore, to make this framework more accessible, a small municipality was used as a case study.

**Figure 7:**
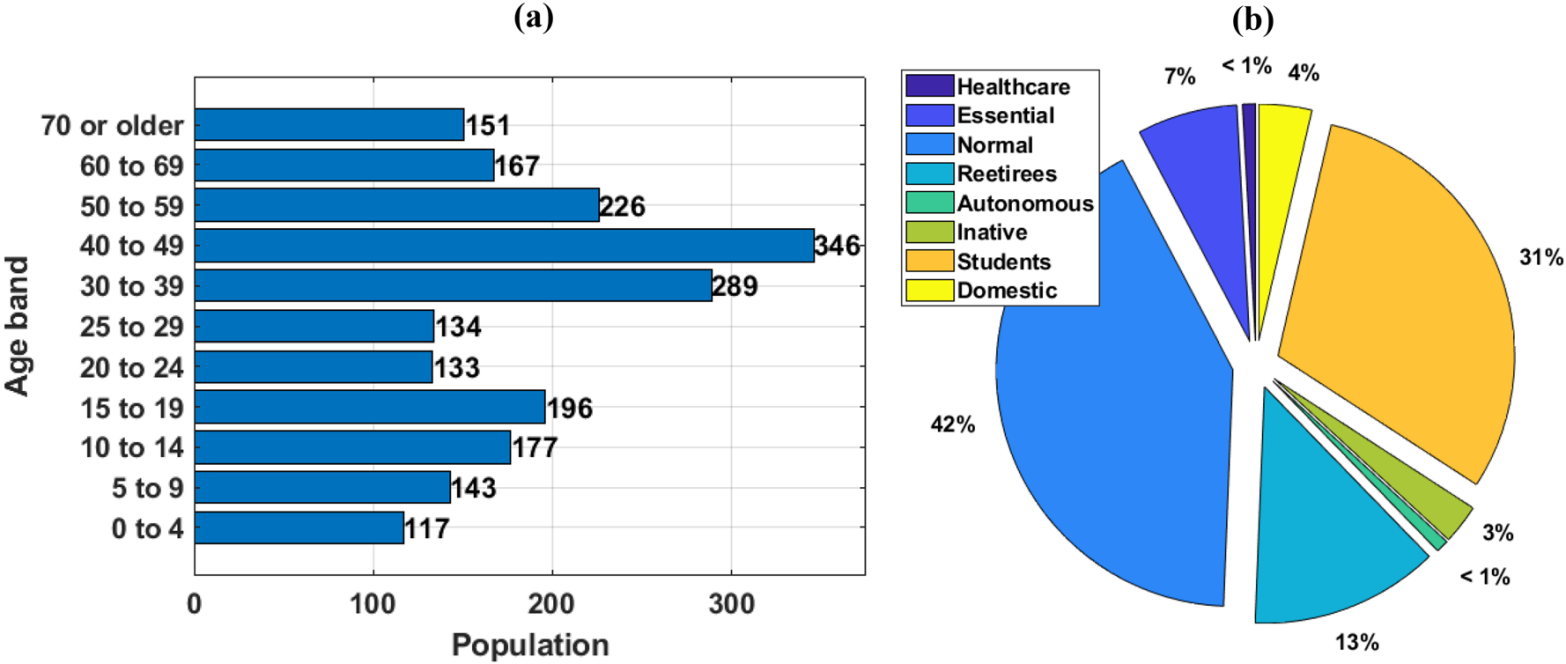
Aged (**a**) and professional activity (**b**) distributions of the Passa Vinte city (MG) city^5^.

Passa Vinte Brazilian city in the Minas Gerais (MG) State, was analyzed by the *MCovidSim-2*. Geographic and Demographic real data of these municipalities were obtained from^5^. In addition, the Pandemic’s real data were obtained from^21^. Table 2 presents geographic and demographic data concerning Passa Vinte (MG). Then a similar way, Fig. 7a,b show the aged and professional activity distribution of this city, respectively.

**Table 2:** Geographic and Demographic data concerning Passa Vinte city^5^.

**Population**		
Estimated population [2021]	2024	People
Population at the last census [2010]	2079	People
Demographic density [2010]	8.43	People/km
**Work and income**		
Average monthly salary of formal workers [2019]	1.6	Minimum salary
Employed population [2019]	15.2	%
**Education**		
Enrollment rate for 6 to 14 year olds [2010]	99	%
Number of elementary schools [2020]	1	Schools
Number of elementary schools [2020]	1	Schools
**Economy**		
GDP per capita [2019]	15598.30	BRL
Municipal Human Development Index (IDHM) [2010]	0.648	
**Health**		
SUS Health Establishments [2022]	3	Facility
Number of ICU [2015]	6	–
**Territory and environment**		
Area of the territorial unit [2021]	246565	km
Adequate sanitary sewage [2010]	59	%
Afforestation of public roads [2010]	62.2	%
Urbanization of public roads [2010]	54.9	%
**Purchase power (refence: minimum wage)**		
No income	564	People
Up to 1/4	99	People
More than 1/4 to 1/2	93	People
More than 1/2 to 1	515	People
More than 1 to 2	335	People
More than 2 to 3	117	People
More than 3 to 5	48	People
More than 5 to 10	36	People
More than 10 to 15	6	People
More than 15 to 20	2	People
More than 30	4	People

Concerning agents’ aspects of mobility, healthy condition, and awareness, data was not found that defines a real distribution and profile of citizens in this analysed city. Therefore, it was assumed that the distribution mentioned in the experiments described in the^6^. Thus, each aspect (agent’s parameters) is defined randomly according to configured distributions. In the end, *MCovidSim-2* constructs the agents’ general matrix that contains all attributes that define society’s behaviour in a given analyzed region (Fig. 1).

Consequently, similar way to^19^, it was assigned a scale from 0 to 100 for the scores according to the aspects attributed to the agents described. Thus, it is known that for higher scores, the agent is more prone to present behaviour that corroborates his infection of the pandemic. Further details can be seen in the work^6^.

## Results and discussions

*MCovidSim-2* was updated using real data from the COVID-19 pandemic from May 26, 2020, to May 26, 2021 (365 days). After determining the sensitive parameters by optimization using the particle swarm algorithm, the *MCovidSim-2* was processed thirty times using the model iteration to the 500th pandemic day, starting from May 26, 2020. In other words, this means evaluating the ability of the updated model to the 365th in forecasting the results from May 26, 2021, onwards. Thus, this procedure defines the model verification task.

After the verification (forecasting) task, the results from the thirty updated model solutions concerning the curve of the cumulative cases were analyzed statistically. A comparison between model results and real data was performed with the boxplot analysis, standard deviation, mean, maximum and minimum model data per epidemiological week. In short, this task defines the updated model’s validation task. It was assumed to apply the social distance policy for all these tasks due to the city’s policy and the real data.

### Analysis of Passa Vinte (MG) city

#### Updating and verification tasks

**Figure 8:**
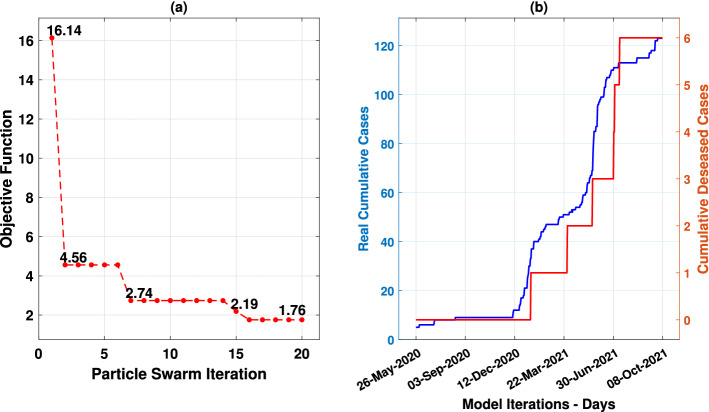
(**a**) Objective function’s convergence result during the processing of the particle swarm algorithm applied for *MCovidSim-2*’s updating using real data from Passa Vinte (MG) city. (**b**) Real cumulative cases and deceased curves.

Given the setup of agent parameters (Fig. 1) and the definition of sensitive parameter intervals (Table 1), the convergence of objective function (Eq. 7) and sensitive parameters results are described in Fig. 8a and Table 3, respectively. Figure 8b shows real cumulative cases (in blue) and deceased (in red) curves of the Passa Vinte (MG) city with the policy distance application.

**Table 3:** *MCovidSim-2*’s sensitive parameters results for the Passa Vinte (MG) city analysis after updating process.

**Sensitive parameters’ results**		
$\beta$	0.464	−
$T_{rec}$	148	Days
$T_{im}$	145	Days
$\gamma$	0.074	−
$Lr_\beta$	0.720	−
$Gr_\beta$	0.513	−
$X_{environment}$	603	−
$X_h$	6.435	% of $X_{environment}$
$X_s$	7.312	% of $X_{environment}$
$X_{ic}$	39.980	% of $X_{environment}$
$X_c$	46.273	% of $X_{environment}$

**Figure 9:**
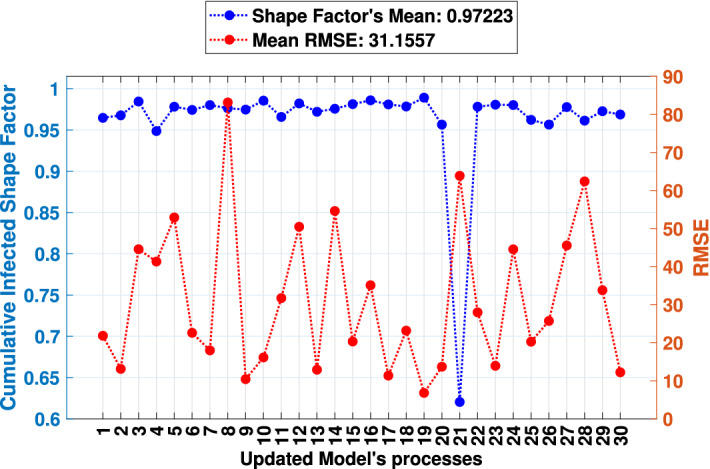
Results of SF (in blue) and RMSE (in red) from thirty times processes of the updated *MCovidSim-2*.

With the updating step completed with the definition of sensitive parameters, the *MCovidSim-2* was processed thirty times to provide data for the verification and statistical analysis tasks of the response prediction from the updated model. Thus, Fig. 9 describes, in blue, the SF of the data curve (Eq. 6) and, in red, the RMSE (Eq. 5) obtained from the comparison of the cumulative cases obtained from model in the thirty obtained solutions with the real cumulative cases results.

Considering model iterations used for updating and forecasting, the obtained overall mean RMSE and SF values for all thirty solutions compared to the real data were 31.15 and 0.97, respectively. Additionally, the 1st, 2nd, 6th, 7th, 9th, 10th, 11th, 13th, 15th, 16th, 17th, 18th to 20th, 22th, 23th, 25th, 26th, 29th and 30th solutions presented the lowest RMSE and are associated with higher SF.

**Figure 10:**
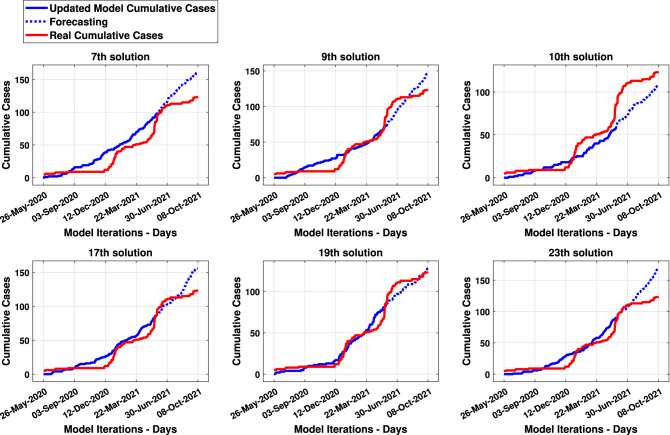
Updated model and real cumulative cases curves—Passa Vinte (MG) analysis.

**Figure 11:**
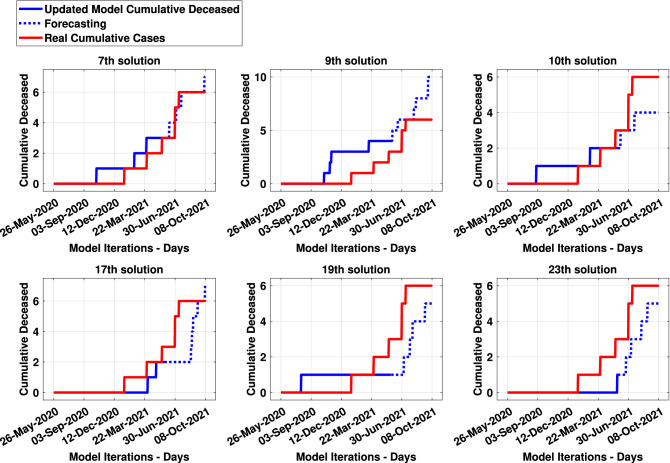
Updated model and real cumulative deceased curves.

Figure 10 presents results and forecasting of the 7th, 9th, 10th, 17th, 19th and 23th solutions as examples. The dotted curve describes the forecast results from 365th to the 500th pandemic day. Concerning the presented solutions previously, Fig. 11 presents the cumulative deceased curves of both the model (in blue) and real data (in red). Similar way, the dotted curve describes the cumulated deceased’s forecasting.

#### Statistical validation task

The twenty solutions mentioned before were considered in the statistical analysis. Other solutions, a priori, were considered outlier responses of the updated model. Nonetheless, the 8th and 21th solutions must be considered due to the high value presented in the RMSE and the low value presented in the SF, respectively. The updated model response variability is due to the agents’ autonomous aspect regarding their movement rules, affecting the spread of the COVID-19 virus directly.

**Figure 12:**
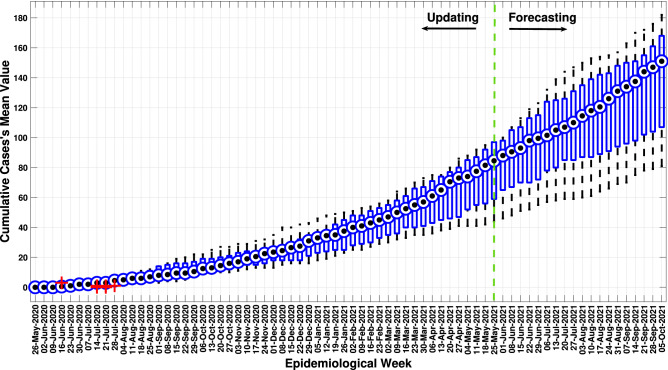
Boxplot analysis of updated model’s response behaviour per epidemiological week.

Thus, the variance study of the model output response behaviour was performed using the boxplot analysis. These responses were compared to the real data concerning cumulative cases over the first day of each epidemiological week (every seven iterations, in a total of 365 model iterations). Figure 12 describes the boxplot graph of the solutions, highlighting the mean value (black dot) obtained from the accumulated number of infected people each week.

**Figure 13:**
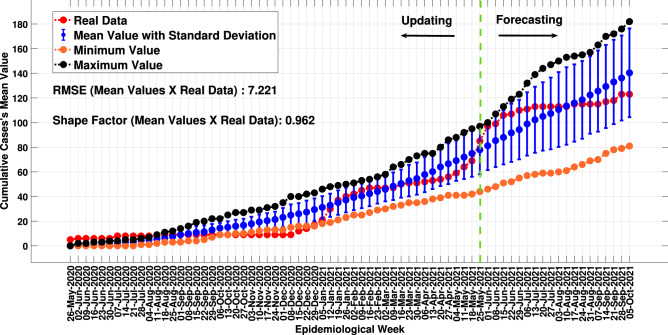
Cumulative infected curve defined by mean values and their comparison with the real data curve.

Note that the solutions obtained show similar response behaviour on the first day of each epidemiological week. Outliers are only observed from June 16, 2020, and July 14, 2020, to August 28, 2020. Additionally, the boxplot analysis provides the mean value of the accumulated infected these days. Therefore, Fig. 13 presents the cumulative infected curve defined by these mean values with its minimum, maximum and standard deviation values from boxplot analysis. Moreover, it also shows its comparison with the real data curve.

Checking the SF and RMSE between the mean solutions’s response curve from the model and the real data (7.22 and 0.96, respectively), the updated *MCovidSim-2* results in the evolution of the cumulative data of infected in the city under analysis consistently. In other words, the model reasonably estimated the cumulative curve of infected up to the 500th day of the pandemic, even performing its update using real data up to the 365th day of the pandemic.

However, the variability of response behaviour throughout the pandemic suggests the continuous need to update the model. For example, the model estimated up to the 500th day; however, it tends to overestimate the cumulative data of infected people beyond the 500th day of the pandemic.

#### Updated model overall results

**Figure 14:**
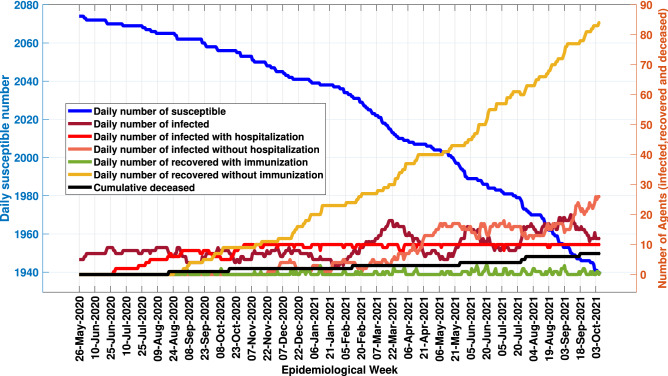
An example of the obtained solution provided by *MCovidSim-2* framework with updated sensitive parameters—19th solution.

After carrying out the updating, verifying and statistical validation steps, *MCovidSim-2* was able to obtain solutions that provide tools for public health administrations’ decision-making process. In the same way that it was possible to know, statistically, the total cumulative number of infected people on the 500th day of the pandemic, it is also possible to know the following predictions: (1) the total number of hospitalized; (2) the total number of infected people who no longer received hospital care due to the limited number of beds available in the region under analysis; (3) the weekly number of infected; (4) application of public policies of social distancing and isolation of specific areas (closing of schools, shops and industries, and restriction of movement)—see Fig. 2; (5) analysis of the natural adaptation (immunization) of the human organism against the virus. Figure 14 presents an example of an obtained solution with the application of the social distancing policy, demonstrating the pandemic’s evolution.

Assuming that the city has the capacity of ten hospital ICUs, it is verified in the 19th solution of the model that the available hospital resources could meet the population’s demand until April 21, 2021. A significant increase in the demand for ICUs (curve of infected people without hospitalization) was identified from this moment. Thus, *MCovidSim-2* can suit as a tool to assist public health management in forecasting the demand for hospital resources needed to serve the population.

**Figure 15:**
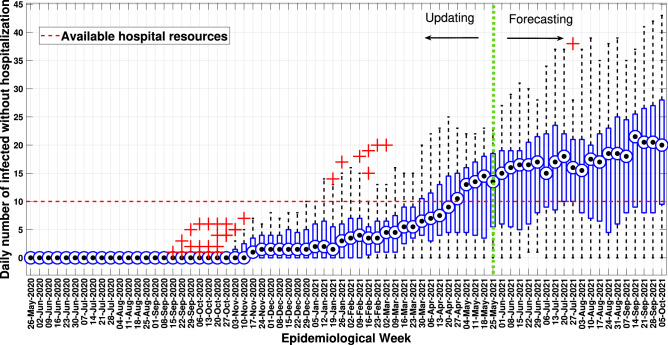
Boxplot analysis of demand for hospital resources.

Therefore, taking the 17 solutions mentioned before, a boxplot analysis was performed regarding the demand for hospital resources. Thus, similarly to what was done to validate the cumulative curve of infected people, Fig. 15 describes the boxplot analysis of this demand, with the average value in each epidemiological week being described (black dot).

Note that due to the presence of outliers, some solutions of the updated model may indicate an overestimation of the demand for hospital resources. This fact can be identified in the epidemiological weeks (iterations) from January 19, 2021, to March 2, 2021. Although this model iteration range belongs to the data used in the update stage, it does not exempt the multi-agent model from providing some inaccuracies or overestimations. Again this is evidenced by the autonomous behaviour of the agents in their movements. A more accurate analysis of hospital demand in the multi-agent approach requires numerous model processing for statistical accuracy in the manager’s decision-making process.

**Figure 16:**
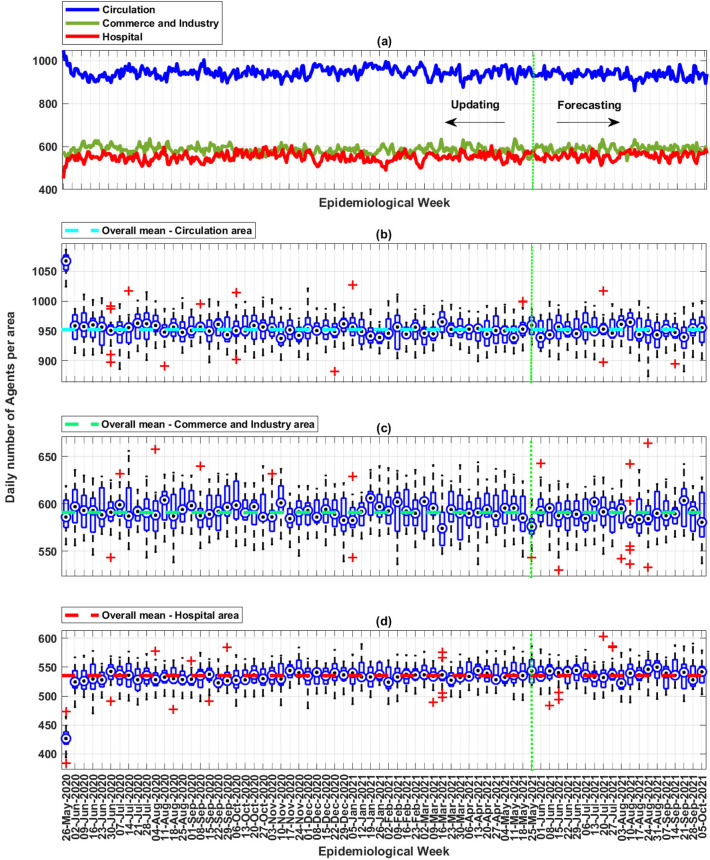
(**a**) Weekly density of agents by specific region with applying the social distancing policy—19th solution. (**b**) Boxplot analysis of all 17 solutions regarding weekly density in the circulation area. (**c**) Boxplot analysis of all 17 solutions regarding weekly density in the commercial and industrial areas. (**d**) Boxplot analysis of all 17 solutions regarding weekly density in the hospital area.

Additionally, the updated *MCovidSim-2* framework provided data regarding agent density by specific environment regions (hospital, school, commerce and industry and; circulation). In this way, the manager can use this functionality to evaluate the application of public policies of social distance and or restrict the movement of people. Therefore, knowing that the update process was based on real data that reflect the social distancing policy applied in the city under analysis, it can be assumed that there were no movements of agents in the area of schools. Figure 16a describes an example of the daily density of agents in the areas of the hospital, commerce and industry and; circulation concerning the 19th solution. Figure 16b–d describe boxplot analysis of the density of agents of all 17 solutions.

In this way, the *MCovidSim-2* framework can provide estimates of the mean daily density of agents in a given region. The general daily means for the city in analysis were 953, 590 and 535 agents in the circulation, commerce and industry, and hospital areas. Additionally, note that the model reflects the population’s behaviour in looking for hospitals in the first weeks of the pandemic.

**Figure 17:**
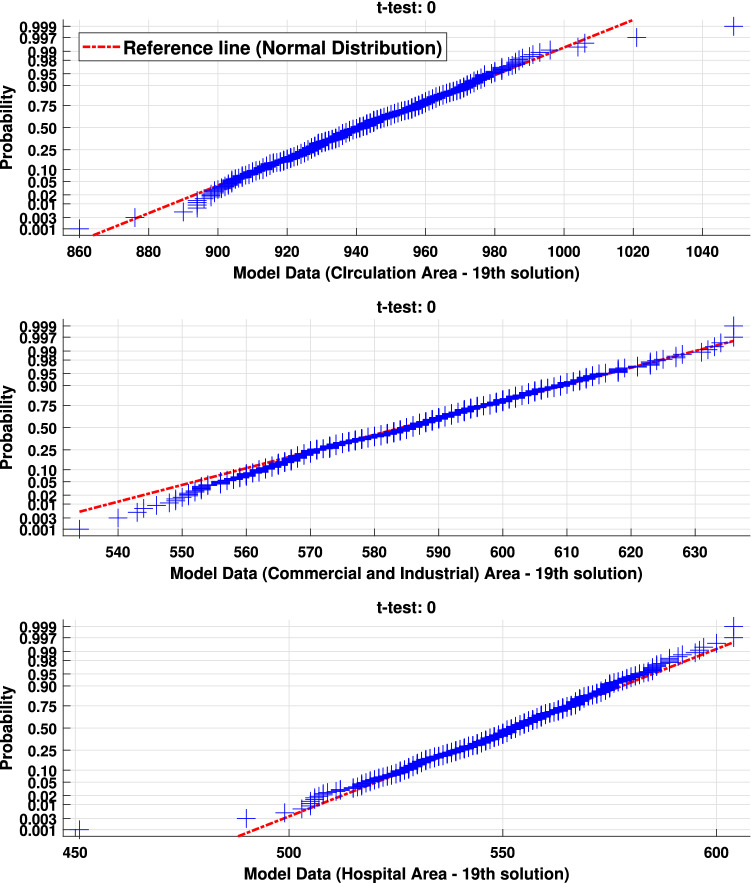
Student’s t-test applied on model data concerning agents’ weekly movement density per environment’s regions (Circulation, Commerce and Industry, and Hospital)—19th solution.

Another aspect identified in the previous figure is that the solutions produce few outliers and evidence of the randomness of the agents’ movement in the environment. To demonstrate this consideration, Fig. 17 presents an example of verification of daily movement density data by applying the *Student’s* *t-test* concerning 19th solution. This test evaluates the null hypothesis that the analyzed data come from a normal distribution around the data mean and unknown variance. This test results in a null value when the hypothesis is true or 1 when this hypothesis can be rejected. In addition, the red line in the graphs of this figure defines the theoretical normal distribution, while the model’s daily density data is represented in blue.

Therefore, the density data respect a normal probability distribution since the blue points appear along the reference line in a confidence interval of 5%.

### Discussions

It could be noted that multi-agent models used to study epidemiological spread in a given society face various challenges and complexities. In several cases, no sufficient data describes social and cognitive aspects like awareness, health conditions, society’s genome, and others. Also, there are cases where the real data does not represent the actual situation due to imprecisions, erroneous medical reports and others situations. Therefore, despite a lack of real data regarding certain aspects of society, many agents’ parameters were needed to implement conceptual models of agents, their behaviour, daily life, and relationships with others into a simulation model.

An updating, verification and validation multi-agent model strategy was formulated using the particle swarm optimization algorithm associated with evaluation metrics such as SF and RMSE. Therefore, these tasks mentioned before were performed to become the epidemiologic model consistently to the real pandemic situation of the Passa Vinte (MG) city by identifying its sensitive input parameters. Then, sensitivity and statistical analysis of the updated *MCovidSim-2* model’s responses was performed using thirty different solutions from the updated model. The boxplot, mean, minimum, maximum, and standard deviation analyses were applied to compare the results with real data.

It could be regarded that the updated model can be leveraged to explore the range of possible solutions in a way that is statistically consistent with known real data and processes. This characteristic can be seen mainly in the verification and validation tasks (Figs. 10, 11, 12, 13) concerning the forecasting of the cumulative model cases. Nonetheless, the updated model overestimates the results after the 500th day of the pandemic. Hence, the multi-agent models for epidemiological system study need to be updated continually due to continuous changes in elementary factors at the individual level that influence emergent system behaviour. These changes can be occasioned, for example, by human natural immunological adaptation, movements behaviour, individual consciousness and other aspects.

Given the tasks of updating, verifying and validating, the framework demonstrated to be a suitable tool to predict the pandemic’s evolution (Fig. 14), providing essential data to the decision-making process of public health policies. Unlike the framework discussed in work by^6^, *MCovidSim-2* furnished forecast data for hospital resources needed to assist the population throughout the evolution of the pandemic (Fig. 15).

Moreover, *MCovidSim-2* also provided data on weekly population density by specific regions (circulation, commerce/industry and hospital). There was no movement of agents to the school’s region due to the social distancing policy applied during the real data acquisition. This functionality of *MCovidSim-2* allows the manager to evaluate policies to restrict population movement in specific areas and regions of the city to mitigate the spread of the virus.

Finally, an implicit modelling aspect that *MCovidSim-2* presents is the consideration of population vaccination effects through the configuration of the parameters of the loss rate ($Lr_\beta$) and the growth rate ($Gr_\beta$) about the probability of contagion of the agent $\beta$. However, this consideration has not been explicitly studied because it is not in the scope of this work.

## Conclusion and recommendations

In this article, a strategy for updating, verifying and updating multi-agent models for studies in epidemiology was introduced. Thus, despite the criticisms and counterpoints regarding the validation process^20^, it can be seen that such models can be suitable for providing a point of view of micro-behaviours and macro-behaviours of the virus dissemination system in a society. This strategy is based on applying the particle swarm optimization algorithm, metrics such as RMSE and SF and sensitivity and statistical analysis to identify the model’s sensitive input parameters.

For this purpose, the framework *MCovidSim-2* based on multi-agents in multi-environments, a modified and improved version of the framework *MCovidSim* discussed in work^6^, was presented to model a real case. Thus, the real data on the pandemic evolution of the Brazilian municipality of Passa Vinte (MG), located in the State of Minas Gerais (MG), were analyzed as a case study.

Despite the complexity of modelling the numerous social and cognitive aspects that describe the behaviour of agents and the difficulties in measuring the dimensions of specific regions (hospitals, schools, commerce and industry; and circulation) of a given city, the updated model of *MCovidSim-2* for the mentioned city was able to: (1) forecast pandemic evolution; (2) provide relevant predictions regarding the management of hospital resources; (3) population immunization adaptation analyzing $Lr_{\beta }$ and $Gr_{\beta }$ rates and i(4) estimate the weekly population density of agents in the circulation, commerce and industry and hospital regions. These forecasting functionalities available in *MCovidSim-2* constitute suitable tools in public health management and the application of social distancing policies.

As a recommendation for future works, this paper motivates study and analysis of the direct vaccination effects and other treatments using the multi-agent approach. Moreover, even knowing the computational cost required, this work motivates the hybridization of the multi-agent approach with machine learning or statistical learning models to create intelligent software agents. This research may allow a profound analysis of the individual real cognitive evolution within society in the face of a pandemic.

### Ethical approval

This article does not contain any studies with human participants or animals performed by any of the authors.

### Informed consent

 As this article does not contain any studies with human participants or animals performed by any of the authors, the informed consent is not applicable.

## Data Availability

The datasets generated and/or analysed during the current study are available in the *MathWorks* repository: https://www.mathworks.com/matlabcentral/fileexchange/118000-m2covidsim2.
